# Preparing linked population data for research: cohort study of prisoner perinatal health outcomes

**DOI:** 10.1186/s12874-016-0174-7

**Published:** 2016-06-16

**Authors:** Lisa Hilder, Jane R. Walker, Michael H. Levy, Elizabeth A. Sullivan

**Affiliations:** National Perinatal Epidemiology and Statistics Unit, University of New South Wales, Sydney, Australia; Faculty of Arts and Social Sciences, University of New South Wales, Sydney, Australia; College of Medicine, Biology and Environment, Australian National University, Canberra, Australia; Faculty of Health University of Technology Sydney, Conjoint School of Women’s and Children’s Health, University of NSW, Sydney, Australia

**Keywords:** Cohort, Linked data preparation, Exposure status, Perinatal, Pregnancy, Prisoner

## Abstract

**Background:**

A study of pregnancy outcomes related to pregnancy in prison in New South Wales, Australia, designed a two stage linkage to add maternal history of incarceration and serious mental health morbidity, neonatal hospital admission and infant congenital anomaly diagnosis to birth data. Linkage was performed by a dedicated state-wide data linkage authority. This paper describes use of the linked data to determine pregnancy prison exposure pregnancy for a representative population of mothers.

**Methods:**

Researchers assessed the quality of linked records; resolved multiple-matched identities; transformed event-based incarceration records into person-based prisoner records and birth records into maternity records. Inconsistent or incomplete records were censored. Interrogation of the temporal relationships of all incarceration periods from the prisoner record with pregnancies from birth records identified prisoner maternities. Interrogation of maternities for each mother distinguished prisoner mothers who were incarcerated during pregnancy, from prisoner control mothers with pregnancies wholly in the community and a subset of prisoner mothers with maternities both types of maternity. Standard descriptive statistics are used to provide population prevalence of exposures and compare data quality across study populations stratified by mental health morbidity.

**Results:**

Women incarcerated between 1998 and 2006 accounted for less than 1 % of the 404,000 women who gave birth in NSW between 2000 and 2006, while women with serious mental health morbidity accounted for 7 % overall and 68 % of prisoners. Rates of false positive linkage were within the predicted limits set by the linkage authority for non-prisoners, but were tenfold higher among prisoners (RR 9.9; 95%CI 8.2, 11.9) and twice as high for women with serious mental health morbidity (RR 2.2; 95%CI 1.9, 2.6). This case series of 597 maternities for 558 prisoners pregnant while in prison (of whom 128 gave birth in prison); and 2,031 contemporaneous prisoner control mothers is one of the largest available.

**Conclusions:**

Record linkage, properly applied, offers the opportunity to extend knowledge about vulnerable populations not amenable to standard ascertainment. Dedicated linkage authorities now provide linked data for research. The data are not research ready. Perinatal exposures are time-critical and require expert processing to prepare the data for research.

**Electronic supplementary material:**

The online version of this article (doi:10.1186/s12874-016-0174-7) contains supplementary material, which is available to authorized users.

## Background

### The MAGIC study

The Mothers and Gestation in Custody (MAGIC) cohort study was set up to assess incarceration effects on pregnancy outcomes [1]. The study used linked records to identify women pregnant while in prison and overcome the lack of pregnancy outcome data for prisoners in the state of New South Wales (NSW), Australia. History of imprisonment is not systematically recorded in pregnancy records. Information about pregnancy is recorded in NSW prison health services paper-based medical records, but this record is not updated with details about the birth or the condition of the baby if the delivery took place after release. Psychiatric illness and substance use were recognised as important confounders of the relationship between incarceration and pregnancy outcomes. Information about these conditions may be available in medical records, but smoking apart, are not included in perinatal data collected at state level in NSW. Serious psychiatric illness and substance use result in inpatient hospital stays and NSW inpatient data includes detailed diagnostic data.

Record linkage had been used elsewhere to obtain information about pregnancy outcomes among prisoners [2, 3]. NSW has appropriate infrastructure to support data linkage: a single computerised record system for managing offenders in the criminal justice system across the state; well-developed state-wide health and vital statistics collections; a jurisdictional register of persons authorised to receive opiate substitution therapy; and, since 2006 a dedicated population health data linkage infrastructure [4]. Dedicated record linkage authorities are increasingly being used to obtain data for observational and health services research [5]. These authorities facilitate the use of linked population data by applying complex population data linkage and the application of best practice principles [6] to protect patient privacy and confidentiality [7]. Researchers are spared the task of linkage, but are responsible for design of the linkage and assessing the quality of the linked data provided to them. NSW accounts for almost one-third of Australia’s births annually [8] and 40 % of the Australian female prisoner population [9].

### The CHeReL

The NSW Centre for Health Record Linkage (CHeReL) is secure linkage facility uses probabilistic methods to link person identifiers extracted from NSW health data collections [10]. The CHeReL promotes the use of linked data by supporting researchers, and works closely with the NSW Population Health Ethics Committee and data custodians. Metadata for these NSW Health data collections are published along with other routinely or commonly linked collections [8].

### The MAGIC data linkage

Five state government-maintained population databases provided data for this study.

1. The **Offender Integrated Management System (OIMS)** is used by Corrective Services NSW to support case management of prisoners aged 18 years or older. Records contain information relating to prisoner location and transfer history, classification, security, self-harm, demographics, and biometric identification. The system was re-organised in 1998 to support routine reporting [11]. Incarceration data for this study excluded police detention, periodic detention and community sentences, but included both women who had been sentenced and women on remand. The OIMS retains all known alternative names, dates of birth and addresses. The extract for data linkage included all known identities.
2. The **Perinatal Data Collection (PDC), previously called the Midwives Data Collection,** is a state wide surveillance system monitoring patterns of pregnancy care, childbirth and newborn outcomes that contains details of all live births and stillbirths of at least 400 g birthweight or at least 20 weeks gestation in NSW [12]. Notification of the birth to the state health authority is a statutory requirement [13]. Each PDC record is unique to a mother-baby pair. Notifications include mother’s names and address and hospital and medical record numbers for both mother and baby. A copy of the form is published [12].
3. The **Admitted Patient Data Collection (APDC)** is an administrative census of services for patients admitted to public and private hospitals, public multi-purpose services, and private day procedure centres in NSW. Each hospital episode record contains information on patient demographics, procedures and diagnoses. Up to 55 diagnoses for each episode are coded using ICD10-AM [14]. From July 2000 the APDC included patient names as mandatory fields for NSW public hospitals, and voluntary fields for private hospitals. All babies, including well babies born alive in NSW hospitals are admitted and assigned a unique hospital record number.
4. The **Pharmaceutical Drugs of Addiction System (PDAS)** is a state-wide register of authorities to prescribe drugs of addiction for opioid substitution therapy (OST). This includes information on the therapeutic substance, the prescriber, and patient demographics. A new authority is issued when there is a change of prescriber or dispensing site. PDAS records retain one alias name in addition to the primary name.
5. The **Register of Congenital Conditions (RoCC)** collates notifications of structural and chromosomal conditions diagnosed during pregnancy and 12 months after birth [12]. Notifications include name and address details for the mother and the child, but these are removed from the register when children reach 5 years of age.

### Linkage by the CHeReL

Person-based record linkage was undertaken by the CHeReL. PDC and APDC are two of the core population health datasets that contribute to the master linkage key (MLK). Each MLK record comprises a unique person number and an encrypted record numbers for each linked record. The MLK is updated each time new data or a new data source is added. Data from other sources, such as OIMS and RoCC can be linked with MLK records. CHeReL generates the project-specific person numbers (PPN) for each linkage that are returned with the relevant encrypted record numbers to the source data custodians. The CHeReL reviews a sample of 1,000 linked project records to assure a false positive rate of ≤0.3 % and a false negative rate of ≤0.5 % the. A report of the linkage was provided to researchers before finalising the linked data [see Additional file 1].

### Linkage design

The MAGIC study set out to examine pregnancy outcomes. PDC records were therefore the primary data source to which all other data were linked. Three data sources added information about maternal history of incarceration, maternal admissions for psychiatric illness, substance use and self-harm and maternal history of OST. The linkage also identified mothers with no history of incarceration or serious mental health morbidity. Two data sources added information about baby outcomes: neonatal admissions; and congenital anomalies diagnosed up to 1 year of age.

PDC records were the primary data source to which all other data were linked. Each PDC record includes identifying data for the mother and the baby. The linkage design specified three steps: (1) linkage of PDC mother data with data from OIMS, APDC mental health admissions and PDAS records; (2) retention of records for all PDC records linked by mother and a random 10 % sample of unlinked PDC mother records; and (3) linkage of records for the babies from the selected PDC records with data from APDC records of neonatal admissions and congenital condition registrations (RoCC). Selection criteria specifying records requested from each collection for data linkage have been included in Table 1.

**Table 1:** Selection of source records and linked records received by researchers for the Mothers and Gestation in Custody (MAGIC) study

	Admitted Patient Data Collection (APDC-M)	Pharmaceutical Drugs of Addiction System (PDAS)	Offender Integrated Management System (OIMS)	Perinatal Data Collection^a^ (PDC)		Admitted Patient Data Collection (APDC-N)	Register of Congenital Conditions^b^ (RoCC)
Selection rules	MH^c^ diagnosis; aged 18–44 years; admitted Jul 00–Dec 06	Authorised for OST^d^ Jan 98–Dec 06; aged 18–44 years	Incarcerated Jan 98–Dec 06; aged 18–44 years	Birth Jan 98–Dec 06; mother aged 18–44 years		Admitted Jul 00–Jan 07; aged 0–28 days	Birth Jan 00–Dec 06
	<-------------Linked by mother -------------->				<------- Linked by baby ------->		
Event	MH^c^ admission	Authority for OST^d^	Incarceration	Maternity^a^	Birth^a^	Neonatal episode	
Selected^e^	230,139	…	…	…	563,547	202,500	
Received^e^	60,464^f^	14,481^f^	9,042^f^	93,284	94,996^f^	37,081^f^	
Person	Woman admitted with MHM	Woman authorised for OST	Prisoner	Mother	Baby	Ill neonate	Baby with anomaly
Selected^e^	81,896	12,526	10,372	404,144	563,547	…	9,945
Received^e^							
Linked	27,511	3,008	3,087	28,973	42,724	32,888	1,384^f^
Unlinked	–	–	–	37,504	52,272	–	–
**Total**	**27,511**	**3,008**	**3,087**	**66,477**	**94,996**	**32,888**	**1,384**

*Notes:*

^a^ PDC birth records are unique to a mother-baby pair

^b^ RoCC were provided as person-based records. Person identifiers are removed from the register after 5 years

^c^ Mental health (MH) admission episodes include one or more ICD10-AM [11] diagnoses of a psychiatric disorder (F00-F09, F20-F99), self-harm (X60-X84, Y10-Y19, Y87.0, Z91.5), drug use (F11-F19, T40, T42, T43), alcohol use (E24.4, F10, G31.2, G62.1, G72.1, I426, K29.2, K70, K86.0, O35.4, R78.0, T51, X45, X65, Y15, Y57.3, Y90, Y91, Z50.2, Z71.4, Z72.1), or flagged as a psychiatric admission

^d^ OST is opiate substation therapy

^e^ The number of records supplied by source data custodians for record linkage were reported to researchers by the CHeReL. Received records were those made available to researchers after linkage or generated from the data received

^f^ The six data sets that were received by researchers

… not available – not applicable

Both OIMS (prisoner) and PDAS (OST authority) data custodians were requested to provide the CHeReL with files containing all permutations of the primary and alias identities.

### Human research ethics committee approval

Ethics approval for the data linkage study was provided by the NSW Population and Health Services Research Ethics Committee (EC00410). Approval for release of prisoner data for linkage was obtained from Justice Health & Forensic Mental Health Network Human Research Ethics Committee (EC00119) and later ratified by the NSW Department of Corrective Services Ethics Committee. Approval to undertake analyses by Indigenous status was obtained from the Aboriginal Health & Medical Research Council Ethics Committee in NSW (EC00342).

### Additional measures to protect privacy

In NSW the provision of health data to researchers about individuals without their consent is conditional on protection from spontaneous recognition of their identities [15, 16]. Additional restrictions are to be expected when the data relates to uncommon and sensitive events such as imprisonment or admissions for psychiatric illness. On advice from data custodians, we did not request dates for key events, but sought instead the age in days of the data subject and the year for all events: birthing; hospital admission; hospital discharge; entry into prison; and release from prison. Further, we agreed to limit the request for population control data to a random unexposed sample rather than whole population data.

### Purpose of the study

The aim of this study was to describe the processing of linked data to make it fit for purpose. This involved data cleaning, preparation of new data to identify incarceration exposure status for each maternity and each mother, identification of the index maternity for each mother and selection of control mothers to enable reassembly of linked data for population research.

## Methods

### Definitions

#### Birth

The event at which a baby of at least 400 g birthweight or at least 20 weeks gestational age is born.

#### Maternity

The event at which a woman gives birth to one baby (singleton birth) or several babies (multiple births).

#### Estimated age at conception

Was calculated as maternal age at birth (days) – gestational age (weeks)*7 + 17. The 17 day correction takes into account that gestational age is measured from the first day of the last menstrual period, which is on average 14 days before conception; and reported as completed weeks, which discounts up to six additional days.

#### Study period

1^st^ July 2000 to 31^st^ December 2006.

#### Incarceration period

1^st^ January 1998 to 31^st^ December 2006.

#### Serious mental health morbidity

APDC record including diagnosis of a psychiatric disorder (F00-F09, F20-F99), self-harm (X60-X84, Y10-Y19, Y87.0, Z91.5), drug use (F11-F19, T40, T42, T43), or alcohol use (E24.4, F10, G31.2, G62.1, G72.1, I426, K29.2, K70, K86.0, O35.4, R78.0, T51, X45, X65, Y15, Y57.3, Y90, Y91, Z50.2, Z71.4, Z72.1) or a flag indicating admission to a psychiatric ward; or PDAS record authorising opiate substitution therapy.

#### Neonatal episode

Hospital episode of a person aged less than 28 days at admission.

### Linked data provided for researchers

Six de-identified data sets were prepared for researchers by source data custodians comprising the PPNs and the study data requested from each source (Table 1).

### Data processing

Five steps were used to process and assemble the linked data:

#### Resolving multiple-matched identities

**Fig. 1:**
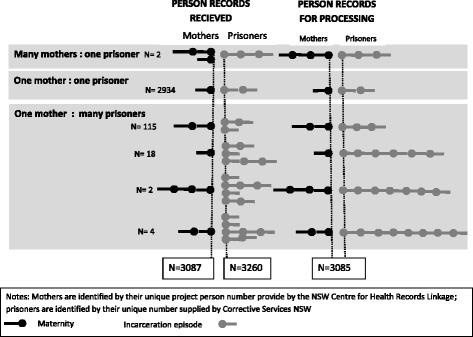
Resolution of multiple matched records

The OIMS Data Custodian provided researchers a ‘unique’ person number (UPN) for each prisoner with the data. Multiple-matched identities were sets of records with one UPN associated with more than one PPN or vice versa, and resolved by assuming each set was truly a single person (Fig. 1) and testing the validity of this assumption with the validation rules. The PDAS data manager resolved records with multiple-matched identities before sending data to researchers.

#### Transformed event-based to person-based records

##### Birth to maternity records

Person-based data can be generated by selecting one event record per person. This simple method, was used to generate maternity data from birth data because only maternal data was required maternal pregnancy outcomes and to check data quality and multiple birth was a planned exclusion factor in subsequent the analysis of baby outcomes. Had information from each baby been needed, the more complex transformation described below, would have been required.

##### Incarceration to prisoner records

A comprehensive person-based record used information from every incarceration event. The event history was important, so these were arranged chronologically. Incarceration order (first, second, etcetera) was added to incarceration records, arranged by episode start age, and the maximum incarceration count per person (N in Table 1) was found. A macro was applied to select and rename the set of selected original or derived data items from each incarceration record to include the event order. The revised incarceration records were then merged by person to form prisoner records consisting of sets of sequentially numbered series of N data items. Thus, 9,042 incarceration records were transformed into 3,087 prisoner records with 30 data items for incarceration start ages (start-age1 start-age2… start-age30), 30 data items for incarceration end ages (end-age1, end-age2 … end-age30), and so forth.

##### Maternity to mother records

Mother records for prisoners were not generated until pregnancy incarceration status for maternities had been assigned (see below).

#### Checks for quality of linked data

The rationale and methods used to identify inconsistences are described below. All maternities for each mother were censored if it was not possible to distinguish between an error in an individual record and a linkage error or the error could affect temporal relationships.

1. **Duplicated birth record**s were identified and removed.
2. **Too many maternities.** It is biologically implausible for a woman to have 15 maternities (Table 1) in 6 and a half years. Mothers with more than one maternity between June and December 2000 or a 3^rd^, 5^th^, 7^th^, 9^th^, 11^th^ and 13^th^ maternity respectively by the end of each successive year were flagged. This conservative rule allowed for the possibility that a woman could give birth twice in 1 year and for repeated preterm birthing.
3. **Non-chronological maternities.** Maternal age in completed years should increase in parallel with the advance in years for successive births. Logical rules were applied to flag records where the number of years of age and the number of calendar years advanced between births differed by more than one.
4. **Concurrent pregnancies.** Conception before or less than 30 days after the previous birth.
5. **Inconsistent incarceration data.** Valid, complete data for the start and end of each incarceration episode was critical to accurate determination of prison pregnancy status.
6. **Conception during incarceration.** Conception in prison is highly unlikely, but not impossible, despite there being a no conjugal visits policy in NSW prisons. Allowance was made for inaccurate dating due to late or no presentation for antenatal care.

#### Assigning pregnancy incarceration status

##### To maternities

The estimated age (days) at conception and the prisoner record was added to each maternity record. Conditional logic was applied to arrays of the ages at the start and end of each incarceration episode and the outcome recorded in a series of a binary (zero or one value) variables were summed to count the number of incarcerations fulfilling each of the following conditions (1) incarceration ended before conception; (2) incarceration started after the birth; (3) incarceration started after conception and ended before the birth; (4) incarceration started after conception and ended after the birth; or (5) incarceration started but had not ended before conception.

Maternities with pregnancy incarceration were those with non-zero counts in categories 3 or 4 (incarceration during pregnancy), while prisoner control maternities had non-zero counts in categories 1 or 2. Maternities with a non-zero count for the final category (conceptions in prison) were censored.

##### To mothers

Maternities for each prisoner mother specifying pregnancy incarceration status were transformed into a prisoner record, which was interrogated to identify pregnant prisoners as those with one or more maternities with a prison pregnancy. Prisoner controls were prisoner mothers with no prison pregnancies. Prisoner mothers with incarceration during pregnancy included a subset with both types of maternity. A flag for prisoner incarceration status was added to each maternity record.

#### Selecting non-incarcerated community controls

The data provided to researchers included birth records for all women with matched incarceration records, all women with matched records for serious mental health morbidity (hospital admission or authority to receive OST) records that included diagnosis of a mental health condition and a 10 % sample of women with no matched records, indicating a history of neither incarceration nor of serious mental health morbidity. The data over-sampled mental health conditions. A population-based random 10 % community control sample comprised the random 10 % sample of mothers with no linked records selected by the CHeReL plus a random 10 % sample of non-prisoner mothers with mental health morbidity whose records had been linked with a record indicting mental health morbidity (Fig. 1).

#### Assigning the index maternity

The index maternity for pregnant prisoners was the first maternity with a pregnancy incarceration. For all prisoner controls and community, the index maternity was the first maternity in the study period.

### Study whole maternity population estimate

An estimate of the number of women aged 18 to 44 years who gave birth in NSW between July 2000 and December 2006 was generated for the study by weighting the validated unlinked control sample count of persons by a factor of 10 and adding the count of validated women with a linked prisoner (OIMS), mental health admission (APDC) or OST authority (PDAS) record.

## Results

### Data validation

#### Alias matching and multiple-matched identities

The CHeReL linkage report [see Additional file 1] noted that 15,995 PDAS identities were supplied for 12,526 women and 64,961 OIMS identities were supplied for 10,372 women. The final linked OIMS records supplied to researchers contained 3,087 different project person numbers (PPNs) and 3,260 OIMS person numbers (UPNs). Fig. 1 summarises the multiple-matched identities: two PPNs each appeared twice, while the same PPN was associated with 2, 3 4 or 5 UPNs in 115, 18, 2 and 4 records respectively.

#### Censored records

Records for 624 women and 1,214 maternities were censored. Of these, records for 578 women were censored because across multiple records their data were inconsistent with being a single individual and 46 because there were no available data to determine temporal relationships between incarceration and pregnancy. Censored women accounted for 0.9 % of all study women, but 16 % of prisoners, 1.7 % of women with mental health morbidity and 0.2 % of non-prisoners with no mental health morbidity (Table 2).

**Table 2:** Reasons for data censoring women by prisoner and mental health morbidity (MHM) status

	Non-prisoners				Prisoners^a^				All MHM^b^		All Prisoners		All study women
	No MHM^b^		MHM^b^		No MHM^b^		MHM^b^						
Person records:	37,533		25,857		935		2,152		28,009		3,087		66,477
Reason censored	*N*	*‰*	*N*	*‰*	*N*	*‰*	*N*	*‰*	*N*	*‰*	*N*	*‰*	*N*
Duplicated births	5	0.1	4	0.2	1	1.1	0	0	4	0.1	1	0.3	10
Too many maternities	8	0.2	6	0.2	1	1.1	7	3.3	308	10.9	8	2.6	22
Non-chronological maternities	25	0.7	29	1.1	4	4.3	26	12.1	55	2	30	9.7	84
Concurrent maternities	42	1.1	37	1.4	6	6.4	20	9.3	57	2	36	11.7	115
Inconsistent incarceration data	–	–	–	–	29	31.0	314	145.9	312	11.1	343	111.1	343
Missing incarceration data	–	–	–	–	18	19.3	41	19.1	41	1.5	59	19.1	59
Conception in prison	–	–	–	–	28	29.3	35	16.3	35	1.2	63	20.4	63
Maternity reason^c^	**64**	**1.7**	**62**	**2.4**	**10**	**10.7**	**42**	**19.5**	**104**	**3.7**	**52**	**16.8**	**178**
Study mothers:	**37,469**		**25,795**		**850**		**1,739**		**27,534**		**2,589**		**65,853**
Censored %	**0.2**		**0.2**		**9.1**		**19.2**		**1.7**		**16.1**		**0.9**

*Notes:*

^a^ Prisoners were also divided into incarceration of 5 days or more (5 + days) or less than 5 days (<5 days). These results are shown in the [see Additional file 2: Table S2A]

^b^ Women with mental health morbidity (MHM) had either a mental health admission episode or were authorised or receive opiate substitution therapy (OST)

^c^ Women censored for inconsistent maternity data have one or more of the first four listed reasons for data censoring

^d^These mothers were retained in the study and their linked records used for further analysis

‰ rate per 1,000 person records % rate per 100 person records – not applicable

Table 2 shows the total number and proportion (per cent) of person records censored and the number and proportion (per 1,000) of persons in each individual censoring category. Some persons had more than one reason for censoring. Inconsistent maternity data applied to all study women, whereas inconsistent incarceration data applied only to prisoners. Women with MHM were over twice as likely (RR 2.2; 95%CI 1.9, 2.6) and prisoners nearly ten times more likely (RR 9.9; 95%CI 8.2, 11.9) to have had their records censored because of inconsistent maternity data than were women with no linked prison or MHM records.

Inconsistent incarceration data was the most common reason overall for censoring, but applied only to prisoner records. Most invalid incarceration data (96 %) were records with incarceration periods that overlapped, the remaining records having inconstant ages (incarceration start ages larger than the end age) or duplicated incarcerations. Multiple matched prisoners (two or more DCSIDs associated with one PPN) accounted for 153 (43 %) of the individuals censored for inconsistent incarceration data. An additional file shows censored records for prisoners with incarcerations lasting less than 5 days and those with one or more periods of incarcerations of 5 or more days [see Additional file 2].

### Maternities with pregnancy incarceration

There were 3,896 maternities in the study period for the 2,589 prisoner mothers included in the study. Of these, 597 maternities with a period of incarceration that coincided with the pregnancy and were further stratified according to incarceration status at the time of giving birthing: 128 maternities with a prison pregnancy where birth took place in prison and 469 where the birth took place in the community after release from prison (Table 3).

**Table 3:** Number of maternities with a pregnancy incarceration, pregnant prisoners and prisoner controls

	Timing of incarceration(s) relative to pregnancy	Pregnant prisoners^a^			Prisoner controls^b^			Total
		Birth out of prison	Birth in prison	Total	Own controls^c^	Peer controls	Total	
Prison maternities	During	166	31	**197**	…	…	…	197
	Before and during	67	9	**76**	185	1,394	1,579	1,655
	After and during	102	44	**146**	134	1,215	1,349	1,495
	Before during and after	134	44	**178**	80	291	371	549
	**Total**	**469**	**128**	**597**	**399**	**2,900**	**3,299**	**3,896**
Prisoner mothers	During pregnancy	166	31	**197**	(113)	–	–	197
	Before and during	67	9	**76**	(45)	1,065	1,065	1,141
	After and during	96	36	**132**	(63)	746	746	878
	Before during and after	116	37	**153**	(65)	220	220	373
	**Total**	**445**	**113**	**558**	**(286)**	**2,031**	**2,031**	**2,589**

Notes:

a Pregnant prisoners have at least one maternity with an incarceration episode during pregnancy. Prisoner mothers can have more than one prison maternity or both prison and non-prison maternities

b Contemporaneous maternities among women incarcerated between 1998 and 2006, but not during pregnancy

c Own control prisoner mothers have both a maternities with pregnancy incarceration and maternity with pregnancy wholly in the community. These prisoner mothers have already been counted with pregnant prisoners

– not applicable () numbers in brackets do not contribute to row total

### Pregnant prisoners and prisoner controls

Pregnant prisoners and prisoner controls are represented by their index maternity in Table 3. The mother-based records identified 558 pregnant prisoners with one or maternities where incarceration coincided with the pregnancy and 2,031 prisoner control mothers with maternities following pregnancies wholly within the community. The 283 prisoners with one or more maternities with a pregnancy incarceration and at one or more maternities with no pregnancy incarceration are presented as ‘Own controls’. This subset of pregnant prisoners did not contribute independently to the total number of prisoners.

### Study population

**Fig. 2:**
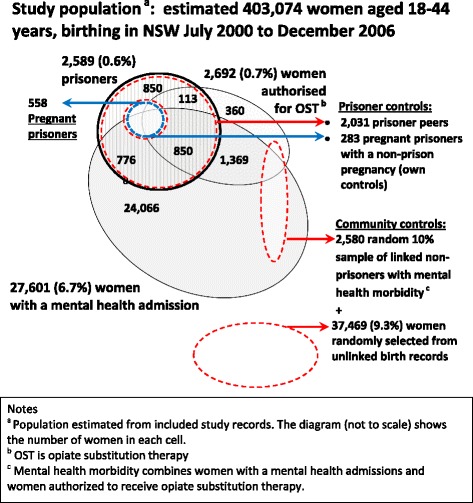
Population prevalence of prison and serious mental health morbidity among childbearing women, NSW July 2000 – December 2006

Figure 2, which is not to scale, shows how the 2,589 prisoner mothers were distributed among study mothers with mental health admissions, mothers authorised to receive OST. Overall the MAGIC study estimated that less than 1 % of 403,047 mothers who gave birth in NSW between July 2000 and December 2006 spent some time in prison between 1998 and 2006. Just over 7 % of the mothers who gave birth were either admitted to hospital with a mental health condition or to a psychiatric ward between July 2000 and December 2006 or were authorised to receive OST between 1998 and 2006 (Fig. 1). The population estimate from final study data represents 99.7 % of the 404,144 women who actually birthed in NSW.

## Discussion

Institutionalised linkage of jurisdictional population data sources is advancing rapidly in Australia [17] and worldwide [18]. This improves the availability and quality of linked data, but the governance and privacy requirements effectively separate researchers from access to the original source data and the linkage process. Researchers are freed from the onerous and highly specialised task of record linkage, but need to specify the linkage design understand the source data, the limitations of the methods used for linkage and consider the likely impacts these could have on the data linked for their research.

NSW Perinatal Data Collection has been audited for the completeness and accuracy of data reported [19, 20] and the coverage has been independently assessed in relation to birth registration data for the state [21]. The quality of hospital episode data are closely scrutinised as these administrative data are the basis for federal funding of state hospitals [22]. There have been several independent studies confirming good linkage between maternity and hospital data in NSW [23–25]. There has been less publicly available information about the quality of corrective services data in NSW, but publication of data from the OIMS suggests confidence in the data quality [11].

Researchers have a responsibility to independently test data quality. Unacceptably high rates of conceptions in prison alerted researchers to the erroneous data from the first linkage and triggered the investigation by Corrective Services NSW and resupply of the data for this research. The CHeReL supported re-linkage. This highlights the importance of good collaborative relationships between linkage authorities, data custodians and researchers.

The use of aliases and the high level of unstable and transient accommodation among people involved with the criminal justice system is common [26, 27] and complicates data linkage [28]. Including alias identities for record linkage of prisoner data increased linkage sensitivity and generated more inclusive sample [29] for a small study population with a relatively high matching prevalence. The MAGIC study was not designed to test the effect of including alias identities on linkage quality. However, there was a substantially higher false positive linkages found among prisoner maternities. This suggests that sensitivity could be compromised for larger studies, particularly where the linkage prevalence is low. This underlines the importance of careful scrutiny of linkage quality when alias identities are used.

Absence of ‘gold standard’ data against which validation could be carried out is a limitation of this study. The data checks carried out were restricted to scrutiny of the data provided. External validation of data linkage requires complex arrangements and resources for investigation of original source records by separate investigators that were not available for this study. However, researchers flagged source records with inconsistent data and provided that these did not breach privacy, returned these to the source data provider. The checks that have been carried out were able to find false linkages, but there is no ready means to identify linkage failure. Available prison statistics in NSW reported cross-sectional data from which it is impossible to assess the number of women who have spent time in prison, let alone how many were pregnant. The MAGIC study was one of the first to use OIMS data for population linkage and heath research.

The MAGIC study produced the first population data from Australia to enable study of the effect of incarceration on pregnancy outcomes [1]. Studies that seek to assess the effect of prison on pregnancy among incarcerated women are relatively sparse because of the difficulties in case finding, the challenges of selecting appropriate comparison groups and the extensive data required to control for socio-economic confounders [2]. This cohort of 597 maternities for 558 pregnant prisoners, of whom 128 gave birth in prison and 2,031 prisoner peers with contemporaneous maternities is one of the largest available series of prison pregnancies. The use of prisoners with contemporaneous pregnancies in the community as a peer control group is a pragmatic and efficient alternative to selecting controls matched on socio-demographic variables.

This was the first data linkage study by the CHeReL to use two-stage matching of PDC data. Mechanisms for dual matching of mother and baby data for perinatal studies have since been formalised [30]. This was also the first CHeReL linkage to use data from the NSW Department of Corrective Services and valuable lessons were learned in the process.

The capacity to report results for prisoners against the whole population increases their utility. The ideal linked population for longitudinal follow-up should include both linked and unlinked data related to the primary exposures for the whole population. Where whole population data cannot be used, and particularly for relatively rare exposures such as female incarceration, a random sample of unlinked data is a pragmatic and effective alternative that can be used to estimate population rates with a high degree of accuracy [31]. The generation an inclusion of pregnancy incarceration status and allocation of each prisoner as either a pregnant prisoner with or without own control status or a prisoner control for validated maternities avoided duplication of effort and provided coherence for all researchers using the data to investigate outcomes.

## Conclusions

Record linkage, properly applied, offers the opportunity to extend knowledge and monitor the effect of interventions aimed at improving health outcomes. Population data linked by dedicated linkage authorities to the highest standard is not research ready and additional effort is needed on the part of researchers to validate and prepare the data for epidemiological analysis.

## Abbreviations

APDC, admitted patient data collection; CHeReL, centre for health record linkage; MAGIC, mothers and gestation in custody; MLK, master linkage key; N, maximum event/episode count per person; NSW, New South Wales; OIMS, offender integrated management system; OST, opioid substitution therapy; PDAS, pharmaceutical drugs of addiction system; PDC, perinatal data collection; RoCC, register of congenital conditions; UPN, unique’ person number provided in prisoner data

## Additional files

Additional file 1. CHeReL linkage summary. This is a copy of the final linkage summary provided to researchers by the Centre for Health Records Linkage (CHeReL) for the MAGIC project. (PDF 112 kb) Additional file 2. Expanded Table S2. This is an expanded version of Table S2 that includes details of prisoner records with incarcerations of less than 5 day’s duration and prisoner records with one or more incarcerations of 5 or more day’s duration. The latter prisoner records were used for the analysis of pregnancy outcomes reported in 2014. (PDF 264 kb)
