# Supplementary material for: Preparing linked population data for research: cohort study of prisoner perinatal health outcomes
Source: BMC Med Res Methodol. 2016 Jun 16;16:72. doi: 10.1186/s12874-016-0174-7 (PMC4910208; doi:10.1186/s12874-016-0174-7)
Supplement: Additional file 1. — CHeReL linkage summary. This is a copy of the final linkage summary provided to researchers by the Centre for Health Records Linkage (CHeReL) for the MAGIC project. (PDF 112 kb) [file 12874_2016_174_MOESM1_ESM.pdf]

Linkage summary

Centre for Health Record Linkage (CHeReL)

Data Linkage. *(Final)*

Project: MAGIC - Mothers and Gestation in Custody, Study of birth and neonatal outcomes of women in prison during pregnancy

METHODS

Sources of data

The Midwives Data Collection (MDC) provides information about the mother's health, pregnancy care and pregnancy outcomes. The data collection covers: up to 2005, all births in New South Wales of at least 400 grams birth weight or 20 weeks gestation; and from 2006, stillbirths of at least 400 grams birth weight or 20 weeks gestation and all live births. The information is recorded by either the midwife or medical practitioner and includes demographic, medical and obstetric information on the mother and information on the labour, delivery and condition of the infant.

The Admitted Patient Data Collection (APDC) includes records for all hospital separations (discharges, transfers and deaths) from all NSW public and private hospitals and day procedure centres. The APDC records include a range of demographic data items (e.g. date of birth, residential address, language spoken at home and country of birth), administrative items (e.g. admission and separation dates) and coded information (e.g. reason for admission, significant co-morbidities and complications and procedures performed during the admission).

The NSW Health pharmaceutical Drugs of Addiction Database (PHDAS) holds personal information and details of authorities to administer methadone or Buprenorphine to nominated individuals.

The Department of Corrective Services Offender Information Management System (OIMS) holds personal, current and previous offence and incarceration data for all persons of 18 years or older who have spent time in prison.

The Birth Defect Register (BDR) provides information about pregnancies affected by a birth defect. The NSW BDR collects clinical and demographic data on pregnancies affected by a defect either structural or chromosomal, birth defects in babies at birth and up to one year of age.

Master Linkage Key

Identifying information such as name, address, date of birth and gender obtained from APDC and MDC Mother records are included in the Master Linkage Key (MLK), which is being constructed by the Centre for Health Record Linkage (CHeReL).[1] No health data are used in this process.

The APDC and MDC Mother records were linked using probabilistic record linkage methods and *ChoiceMaker* software.[2] *ChoiceMaker* uses 'blocking' and 'scoring' to identify definite and possible matches. During blocking, *ChoiceMaker* searches the target datasets for records which are possible matches to each other. There are two types of blocking. The exact blocking algorithm requires records to have the same set of valid fields and the same values for these fields. The automated blocking algorithm builds a set of conditions that are used to find as many as possible records that potentially match each other. Scoring employs a combination of a probabilistic decision, which is computed using a machine learning technique, and absolute rules, which include upper and lower probability cut-offs, to determine the final decision as to whether each potential match denotes or possibly denotes the same person. Upper and lower probability cut-offs initially start at 0.75 and

0.25 for a linkage and are adjusted for each individual linkage to ensure false links are kept to a minimum.

At the completion of the process, each record in the MLK is assigned a record identification number and a MLK person ID to allow linked records for the same individual to be identified and extracted.

Summary of results:

Mother's Case probabilistic record Linkage produced 28,973 mothers having 42,724 babies. These 28,973 mothers linked to 60,464 APDC records, 26,261 OIMS records and 3,992 PHDAS records. Then a sample 10% of the remaining Mothers was taken by getting a list of all mothers in the period 1/7/2000 to 31/12/2006 and taking away mothers who had been included in the Case linkage. There were 375,040 mothers remaining after taking away mothers included in case linkage, and from these a random sample of 37,504 mothers was generated. Then all MDC mother records were extracted for those sample mothers (52,272 records).
These Mother MDC records were sent back to NSW Health to provide a list of 99,772 MDC baby records that matched to these mothers. The CHeReL then produced a probabilistic record Linkage of MDC Baby records and APDC records (that matched to MDC baby records) to BDR records. The result of this linkage was 34,043 MDC baby records matched to 37,899 APDC records and 1,528 BDR records. 65,729 MDC baby records were unmatched.

Summary Mothers Linkage:

1) Extract of MDC Mothers and APDC records from CHeReL Master Linkage Key (MLK).

A set of APDC record IDs was supplied by the NSW Department of Health for Females who were 18 to 44 and admitted with an ICD10AM diagnosis of drug or alcohol use or Psychiatric disorders in the period 1/7/2000 to 31/12/2006. These APDC records were used to extract (using the CHeReL MLK) MDC mother records that had one or more Births in the period 1/7/2000 to 31/12/2006. . *(Refer Table 1)*

Results: 230,139 APDC records were provided by NSW Health and 42,724 MDC Mother records were extracted from the CHeReL Master Linkage Key which linked to 60,464 of the subset ADPC records provided. This equated to 27,511 persons. *(Refer Table 2)*

2) Adhoc Linkage of (MDC Mothers + APDC records) to (OIMS + PHDAS) records.

The following NSW Government Departments provided the CHeReL with Encrypted Record Identifiers and Demographic data:
i) The NSW Department of Corrective Services extracted OIMS records for females aged 18-44 that spent time in prison between 1/1/1998 and 31/12/2006. *(Refer Table 1)*
ii) The Pharmaceutical Services Branch of NSW Health extracted records for all females aged 18-44 at admission of treatment between 1/1/1998 and 31/12/2007. . *(Refer Table 1)*

These Two datasets were linked using probabilistic record linkage to the MDC + APDC records.

Results:

The 103,188 CHeReL MLK records (Obtained in step 1) linked to 3,992 PHDAS records (3,056 person Ids) and 26,261 OIMS records (3,087 person IDs). *(Refer Table 2)*

3) Selection of 10% of unmatched MDC Mothers.

A list of all persons that contain MDC mother records for the period 1/7/2000 to 31/12/2006 was created and persons that were matched to a APDC, OIMS, or PHDAS records were removed. Then a random 10% sample was taken of the remaining CHeReL MLK Persons and the MDC Mother records were extracted for that person.

Results: There were 404,013 persons that contained a MDC Mother record. (using same periods and ages as matched MDC Mother data). The 28,973 persons that matched to APDC or OIMS or PHDAS were deleted, leaving 375,040 non-matching Persons. A random 10% sample was taken (37,504 persons). All MDC mother records for the sample persons in the period 1/7/2000 to 31/12/2006 (52,272 MLK records) were then extracted from the CHeReL MLK.)

Summary Babies Linkage:

1) Extract of MDC Babies and APDC records from CHeReL Master Linkage Key (MLK).

A set of 99,772 MDC baby record IDs was supplied by the NSW Department of Health for all mothers selected for this study. These MDC baby records were used to extract (using the CheReL MLK) APDC records that were included in the neonatal subset also supplied by NSW Health*. (Refer Table 3)*

Results: 198,708 APDC records were provided by NSW Health along with 99,772 MDC Baby records. Records were extracted from the CHeReL MLK for all MDC baby records and APDC records that matched to the 99,772 MDC baby records. There were 37,899 APDC records that matched to MDC baby records. *. (Refer Table 4)*

2) Adhoc Linkage of (MDC Babies + APDC records) to BDR records

The BDR dataset provided by NSW Health was linked using probabilistic record linkage to the MDC baby records + APDC records.

Results: 37,899 APDC records and 99,772 MDC baby records were linked to 9,945 BDR records to give 1,528 BDR records that linked to a MDC baby record. (Refer table 4)

Error Rates:

The CHeReL Master Linkage Key is regularly checked for false positive linkages.

A random sample of 1000 Person IDs was selected from the Master Linkage Key (2009_07a) used to select records to be provided to the study investigators and reviewed for false positive linkages:

False positive rate = 3/1,000 records (0.3%)
False negative rate <5/1,000 records (< 0.5%)

Table 1: Data sources and record types (Mothers Data)

Data Source	Description
Midwives Data Collection (MDC) - Mothers<br>**[Master Linkage Key 2009_07a]**	All MDC Mother records with a Baby's Date of Birth from 1 July 2000 to 31 Dec 2006<br>Mother was 18-44 years old at Baby's Date of Birth<br>Total = 563,547 records (404,144 Persons)
Admitted Patient Data Collection (APDC)<br>**[Master Linkage Key 2009_07a]**	Episodes of care selected for the following parameters:<br>All females aged 18-44 and admitted with following diagnosis codes:<br>Psychiatric illness F00-F09, F20-F99<br>Self Harm (Incl poisoning) X60-X84, Y10-Y19, Y87.0, Z91.5<br>Drug Use F11-F19, T40, T42, T43<br>Alcohol Use E24.4, F10, G31.2,G62.1, G72.1, I426, K29.2, K70, K86.0, O35.4, R78.0, T51, X45, X65, Y15, Y57.3, Y90, Y91, Z50.2, Z71.4, Z72.1<br>From 1 July 2000 to 31 Dec 2006<br>Total = 230,139 records (81,896 Persons)
NSW Pharmaceutical Drugs of Addiction Database (PHDAS)	selected for the following parameters:<br>Females aged 18-44 at admission of treatment between 1/1/1997 and 31/12/2007<br>Total = 15,995 records (12,526 Persons)
NSW Department of Corrective Services<br>(OIMS database)	selected for the following parameters:<br>Females aged 18-44 that spent time in prison between 1/1/1998 and 31/12/2006<br>Total = 64,961 records received from DCS<br>(10,372 Persons)

Table 2: Summary of records (Mothers) returned to Study Investigators

Measure	Record type	No.
Total All Mother Records to be returned to Study:	Total Matched records<br>**Total Project Person Numbers (PPN)**	133,441<br>**28,973**
	UnMatched MDC Mother records 10% Sample of all Un-matched MDC mother persons:<br>(total persons unmatched: 375,040 persons)	52,272
	**Project Person Numbers (PPN):**	**37,504**

Matched MDC Mother records	MDC Mother records that matched to any APDC or OIMS or PHDAS records:<br>Project Person Numbers (PPN)	42,724<br>28,973
Matched APDC records	Subset APDC records that matched to MDC Mother record:<br>Project Person Numbers (PPN)	60,464<br>27,511
Matched OIMS records	OIMS records that matched to MDC Mother record:<br>Project Person Numbers (PPN)	26,261<br>3,087
Matched PHDAS records	PHDAS records (Patientids) that matched to MDC Mother record:<br>Project Person Numbers (PPN)	3,992<br>3056

Table 3: Data sources and record types (Babies Data)

Data Source	Description
Midwives Data Collection (MDC) - Babies<br>**[Master Linkage Key 2009_07a]**	All MDC Baby records that matched with Mothers MDC records selected from Mothers Linkage in table 2<br>In period from 1 July 2000 to 31 Dec 2006<br>Total = 99,772 records (99,758 Persons)<br>(there were 14 Babies who had duplicate MDC records when matching demographic information)
Admitted Patient Data Collection (APDC)<br>**[Master Linkage Key 2009_07a]**	Episodes of care selected for the following parameters:<br>All neonatal records for admissions of Babies aged 0-27 days with following diagnosis codes:<br>(P00.0 - P99.8)<br>For period from 1 July 2000 to 31 Dec 2006<br>Total = 198,708 records
NSW Birth Defects Register. (BDR)	selected for the following parameters:<br>Records for the Period between 1/1/2002 and 31/12/2006<br>Total = 9,945 records

Table 4: Summary of records (Babies) returned to Study Investigators

Measure	Record type	No.
Total All Baby Records to be returned to Study:	Total Matched records<br>**Total Project Person Numbers (PPN)**<br><br>UnMatched MDC Baby records :<br>**Project Person Numbers (PPN):**	73,470<br>**34,039**<br><br>65,729<br>**65,719**
Matched MDC Mother records	MDC Mother records that matched to any APDC or BDR records:<br>Project Person Numbers (PPN)	34,043<br>34,039
Matched APDC records	Subset APDC records that matched to MDC Baby record:<br>Project Person Numbers (PPN)	37,899<br>33,368
Matched BDR records	BDR records that matched to MDC Baby record:<br>Project Person Numbers (PPN)<br>(there were 3 Babies who had duplicate BDR records when matching demographic information)	1,528<br>1,525

References

1. The Centre for Health Record Linkage at: www.cherel.org.au.
2. ChoiceMaker Technologies, Inc. New York, NY 10010
