# Supplementary material for: Preparing linked population data for research: cohort study of prisoner perinatal health outcomes
Source: BMC Med Res Methodol. 2016 Jun 16;16:72. doi: 10.1186/s12874-016-0174-7 (PMC4910208; doi:10.1186/s12874-016-0174-7)
Supplement: Additional file 2. — Expanded Table S2. This is an expanded version of Table S2 that includes details of prisoner records with incarcerations of less than 5 day’s duration and prisoner records with one or more incarcerations of 5 or more day’s duration. The latter prisoner records were used for the analysis of pregnancy outcomes reported in 2014. (PDF 264 kb) [file 12874_2016_174_MOESM2_ESM.pdf]

Table 2A: Reasons for data censoring women by prisoner and mental health morbidity (MHM) status

	Non-prisoners				Prisoners [a]								All		All		All study women
	No MHM [b]		MHM [b]		No MHM [b]		MHM [b]		< 5 days		5+ days		MHM [b]		Prisoners		
**Person records:**	**37,533**		**25,857**		**935**		**2,152**		**1,048**		**2,039**		**28,009**		**3,087**		**66,477**
**Reason censored**	*N*	‰	*N*	‰	*N*	‰	*N*	‰	*N*	‰	*N*	‰	*N*	‰	*N*	‰	***N***
Duplicated births	5	0.1	4	0.2	1	1.1	0	0	0	0	1	0.5	4	0.1	1	0.3	**10**
Too many	8	0.2	6	0.2	1	1.1	7	3.3	1	1	7	3.4	308	10.9	8	2.6	**22**
Non-chronological	25	0.7	29	1.1	4	4.3	26	12.1	3	2.9	27	13.2	55	2	30	9.7	**84**
Concurrent	42	1.1	37	1.4	6	6.4	20	9.3	8	7.6	18	8.8	57	2	36	11.7	**115**
Inconsistent	–	–	–	–	29	31.0	314	145.9	4	3.8	339	166.3	312	11.1	343	111.1	**343**
Missing	–	–	–	–	18	19.3	41	19.1	3	2.9	56	27.5	41	1.5	59	19.1	**59**
Conception in prison	–	–	–	–	28	29.3	35	16.3	0	0	63	30.9	35	1.2	63	20.4	**63**
**Maternity reason [c]**	**64**	**1.7**	**62**	**2.4**	**10**	**10.7**	**42**	**19.5**	**10**	**9.5**	**42**	**20.6**	**104**	**3.7**	**52**	**16.8**	**178**
**Study mothers :**	**37,469**		**25,795**		**850**		**1,739**		**1,031**		**1,558**		**27,534**		**2,589**		**65,853**
**Censored %**	0.2		0.2		9.1		19.2		1.6		23.6		1.7		16.1		0.9

Notes:

[a] Prisoners were divided into two groups; with/without mental health morbidity (MHM); or incarceration of 5 days or more (5+days) or less than 5 days (<5 days).

[b] Women with mental health morbidity (MHM) had either a mental health admission episode or were authorised or receive opiate substitution therapy (OST).

[c] Women censored for inconsistent maternity data have one or more of the first four listed reasons for data censoring

‰ rate per 1,000 person records % rate per 100 person records – not applicable
